# Using Mobile EEG to Investigate Alpha and Beta Asymmetries During Hand and Foot Use

**DOI:** 10.3389/fnins.2020.00109

**Published:** 2020-02-14

**Authors:** Julian Packheiser, Judith Schmitz, Yaolu Pan, Yasmin El Basbasse, Patrick Friedrich, Onur Güntürkün, Sebastian Ocklenburg

**Affiliations:** ^1^Institute of Cognitive Neuroscience, Biopsychology, Department of Psychology, Ruhr-University Bochum, Bochum, Germany; ^2^School of Medicine, University of St Andrews, St Andrews, United Kingdom; ^3^Brain Connectivity and Behaviour Laboratory, Sorbonne Universities, Paris, France; ^4^CNRS, Groupe d’Imagerie Neurofonctionnelle, Institut des Maladies Neurodégénératives, UMR 5293, CEA, University of Bordeaux, Bordeaux, France; ^5^Department of Psychology, University of Duisburg-Essen, Essen, Germany

**Keywords:** laterality, oscillations, asymmetry, EHI, WFQ, Edinburgh Handedness Inventory, Waterloo Footedness Questionnaire

## Abstract

The Edinburgh Handedness Inventory (EHI) and the Waterloo Footedness Questionnaire (WFQ) are two of the most widely used questionnaires to assess lateralized everyday behavior in human participants. However, it is unclear to what extent the specific behavior assessed in these questionnaires elicit lateralized neural activity when performed in real-life situations. To illuminate this unresolved issue, we assessed EEG alpha and beta asymmetries during real-life performance of the behaviors assessed in the EHI and WFQ using a mobile EEG system. This methodology provides high ecological validity for studying neural correlates of motor behavior under more naturalistic conditions. Our results indicate that behavioral performance of items of both the EHI and WFQ differentiate between left- and right-handers and left- and right-footers on the neural level, especially in the alpha frequency band. These results were unaffected by movement parameters. Furthermore, we could demonstrate that neural activity elicited specifically during left-sided task performance provides predictive power for the EHI or WFQ score of the participants. Overall, our results show that these prominent questionnaires not only distinguish between different motor preferences on the behavioral level, but also on the neurophysiological level. Furthermore, we could show that mobile EEG systems are a powerful tool to investigate motor asymmetries in ecologically valid situations outside of the laboratory setting. Future research should focus on other lateralized behavioral phenotypes in real-life settings to provide more insights into lateralized motor functions.

## Introduction

Most humans consistently prefer one hand or one foot over the other in everyday life (Porac and Coren, 1981; Güntürkün and Ocklenburg, 2017). Both human handedness and footedness are among the most well-studied lateralized behaviors. A recent meta-analysis involving a sample of over 2 million participants found that 81.9% of the population is right-handed using a non-right/right classification (Papadatou-Pastou et al., 2019). For footedness, the prevalence of lateralized preferences in the population is less clear as no large-scale meta-analysis exists as of yet. A substantial sample of 3307 participants was investigated regarding their footedness by Coren (1993). He found that 86.7% of all participants were right-preferent, 7.1% were left-preferent, and 6.2% had no foot preference. This finding effectively demonstrates a similar distribution as was identified for handedness. These measures are generally positively correlated in individuals supporting this finding (Porac and Coren, 1981; Brown and Taylor, 1988; Reiss et al., 1999; Dittmar, 2002; Ocklenburg and Güntürkün, 2009).

A major interest in studying these lateral biases derives from the association between lateralized motor behavior with lateralized cognitive domains like language (Ocklenburg et al., 2014) and emotional processes (Elias et al., 1998) but also psychopathologies such as schizophrenia (Sommer et al., 2001; Dragovic and Hammond, 2005; Ocklenburg et al., 2015 for handedness; Tran et al., 2015 for footedness). In modern laterality research, handedness and footedness are usually assessed as continuous measures using questionnaires rather than dichotomous measures such as asking about the “writing hand” (as e.g., in Turnbull et al., 2001) or “kicking foot” (as e.g., in Brown and Taylor, 1988). Although dichotomous measures are seemingly plausible strategies to assess limb preferences, they come with certain disadvantages. Using for example “writing hand” as a measure for handedness results in a handedness mismatch of only about 0.4% for right-handers, but left-handers are falsely classified as right-handers in 13.5% of the cases because many left-handers use their right hand for writing (Papadatou-Pastou et al., 2013). Furthermore, the forced-choice nature of this assessment does not allow for mixed-handedness as a result. Therefore, continuous measures possess considerable advantages compared to dichotomous measures because they allow more accurate phenotyping and capture more individual variance, which is essential for understanding the underlying neural mechanisms of lateralized behavior (Kanai and Rees, 2011). Two questionnaires arose as the standard measures of human limb asymmetries:

For handedness, the most cited questionnaire is the Edinburgh Handedness Inventory (EHI, Oldfield, 1971) with well over 20,000 citations (Google Scholar). It has been identified to be the most widely used handedness questionnaire (Fazio et al., 2012). The EHI comprises 10 items of everyday tasks (writing, throwing a ball, drawing, using scissors, brushing teeth, using a knife, using a spoon, using a broom, striking a match and opening a box-lid/jar) and instructs the participants to assess their hand preference for each of them by entering a “ + ” sign either in a left or right preference column. If these hand preferences are so strong that the participants would never use the other hand for this task unless being absolutely forced to, they are instructed to enter a “ + + ”. If there is no preference for a specific task, the participants are instructed to enter a “ + ” in both the left and right preference column. For footedness, one of the most prominent questionnaires is the Waterloo Footedness Questionnaire (WFQ, Elias et al., 1998). Here, participants are asked to indicate whether they would rather perform everyday mobility and stability tasks (ball kicking, hopping on one leg, standing on one foot, smoothing sand, stepping onto a chair, weight-shifted relaxed standing, stepping on a shovel, grasping a marble, balancing on a rail and stepping on a bug) with the left or with the right foot/leg. Similar to the EHI, the answers can indicate the consistency of the foot/leg preference (whether they preferably use the left or right foot). Therefore, the EHI and WFQ are highly comparable as items in both questionnaires have five different outcomes (strong leftward preference, weak leftward preference, no preference, weak rightward preference, and strong rightward preference) and the evaluation of both questionnaires is represented in form of a laterality quotient.

While evaluative studies indicate both the EHI and WFQ to yield reliable measures that are relatively stable over time (Ransil and Schachter, 1994; Camargos et al., 2017), there has been some dispute about their validity to measure handedness or footedness respectively. Due to its broad usage, some studies have investigated the factorial validity of the EHI to determine how well its individual items measure handedness. Using exploratory factor analysis, Bryden (1977) identified that there were inconsistencies between three items of the questionnaire among right-handers, namely the opening of a box/jar, using a broom and the striking of a match. McFarland and Anderson (1980) found supporting results as using a broom and box/jar opening again poorly loaded on a converged handedness factor. Furthermore, they found the same result to be true for using a knife. This finding is contrasted by large-scale latent variable analysis that found the usage of a knife to be one of the best predictors for handedness (Tran et al., 2014).

The factorial validity was also investigated using confirmatory factor analysis. Here, it was again confirmed that broom usage and box/jar opening demonstrated considerable error variance rates indicating against their validity to assess handedness (Dragovic, 2004; Milenkovic and Dragovic, 2013). Veale (2014) even found that further removing items on scissor and knife usage as well as striking a match provided a better model fit indicating that these items do not load well on a single handedness factor. However, given its widespread use in the literature, the application of the EHI allows for comparability between studies, giving the EHI an advantage over other questionnaires.

Given the far less widespread prevalence of studies investigating footedness in general, it is unsurprising that the items of the WFQ have not been investigated with such rigor as is the case for the EHI. Up until now, factorial validity of the WFQ has only been compared between the mobility and the stability subscales of the questionnaire (Kapreli et al., 2015). Since the study found high correlations between the two subscales, it was concluded that the WFQ subscales measure a single footedness factor. While the two subscales seem to measure similar constructs of footedness, a specific investigation of individual items on the mobility subscale has not supported a one-dimensional measure of footedness. Tran and Voracek (2016) used psychometric measures in a well-powered sample and could show that a two-dimensional rather than a one-dimensional model provides a much better fit to the data for items that are also used in the WFQ. Thus, individual items of the WFQ do not seem to load on a single footedness factor on the behavioral level.

Both questionnaires have in common that their reliability and their factorial validity have been measured using the behavioral output of the participants. However, no study has so far investigated the validity of individual items of the EHI or the WFQ on the neural level. Therefore, the aim of the present study was to identify the neurophysiological validity of both questionnaires using mobile electroencephalography techniques. Mobile EEG systems enable to record brain activity during active movement thus allowing for the measurement of highly ecologically valid neurophysiological signals. Until recently, research on the neural correlates of motor behavior relied mostly on artificial settings investigating, e.g., finger tapping tasks with low ecological validity using stationary EEG or fMRI systems (e.g., Turesky et al., 2018; Schmitz et al., 2019). Mobile EEG systems tackle these shortcomings by allowing for more naturalistic behavior during physiological recordings (Gramann et al., 2014). However, only a handful of studies have been published so far using this novel technology (e.g., Griffiths et al., 2016). We used mobile EEG to identify how well-individual items of both the EHI and the WFQ differentiate between left and right body movements both in left- and right-handers and left- and right-footers on the neural level. It is well-established how the motor cortex controls behavioral output of the limbs (Kim et al., 1993; Hammond, 2002), namely that the left hemisphere dominantly controls the right body side and that the right hemisphere dominantly controls the left body side. Using stationary EEG systems, studies have reported increased asymmetries over sensorimotor electrodes in the alpha and beta frequency bands during unilateral movements (Pfurtscheller, 1981; Deiber et al., 2012). Therefore, we hypothesize that alpha and beta power asymmetries differentiate between left- and right-handers and left- and right-footers during task performance of EHI and WFQ items. Furthermore, we hypothesize that activity in these two frequency bands allows for a differentiation between left and right performance of these items.

## Materials and Methods

### Participants

A total of 51 participants (32 females) took part in this study. The age range was between 18 and 34 years (mean age = 25.46 years, *SD* = 3.59 years). Handedness of the participants was assessed in a pre-screening using the EHI. 26 of the participants were consistently left-handed (EHI lateralization quotient < −40, mean = −87.42, *SD* = 15.84) and 25 of the participants were consistently right-handed (EHI lateralization quotient > + 40, mean = 90.03, *SD* = 12.70). Mixed-handed participants were excluded during the pre-screening process. Lateralization quotients (LQs) were calculated using the following formula: LQ = [(R-L)/(R + L)]^∗^100, with R indicating the number of right preferred tasks and L the number of left preferred tasks of the EHI items. The cut-offs for left-handedness and right-handedness (< −40 and > + 40, respectively) were derived from previous studies (Li et al., 2003) based on findings linking handedness to cognitive abilities (Burnett et al., 1982). There were no sex differences in EHI scores [*t*(50) = 1.13, *p* > 0.250]. Furthermore, left- and right-handers did not differ in age [*t*(50) = 0.06, *p* > 0.250]. All participants had normal or corrected-to-normal visual acuity. Participants with known neurological or psychiatric disorders were excluded from the study. The study was conducted in accordance with the declaration of Helsinki and was approved by a local ethics committee of the psychological faculty at Ruhr-University Bochum. All participants gave written informed consent.

### Experimental Task

The experimental paradigm consisted of two sessions, one for items of the EHI (conducted first) and one for items of the WFQ (conducted second):

In the EHI session, participants were instructed to perform the 10 tasks mentioned in the EHI both with the left and with the right hand. Each task was performed for 1 min per hand. Thus, the EHI session consisted of 20 trials (10 tasks, once per hand) and the trials were separated by an ITI of 30 s. After the ITI, participants were informed about the task of the following trial and with which hand it had to be performed via a standardized oral instruction. Left and right trials of the identical task were always conducted consecutively. The order of the tasks was randomized and counterbalanced across participants to exclude any serial order or exhaustion effects. Furthermore, we also randomized the starting side (left or right) for each individual task to exclude potential effects due to experience with the respective task. The experimenter assisted in handing the participants the necessary equipment for each specific trial. The tasks were executed as follows:

(1) Writing: the participants wrote down letters of the alphabet from a to z (restarting from the beginning of the alphabet after reaching z)
(2) Throwing: the participants threw a ball against a wall with the experimenter picking up and handing the ball to the participant
(3) Drawing: the participants drew along a pre-designed sketch
(4) Scissors: the participants cut out a spiral shape holding the scissor with one hand (instructed hand) and the piece of paper with the other
(5) Brushing teeth: the participants brushed their teeth
(6) Knife: the participants cut a piece of clay
(7) Spoon: the participants simulated eating soup from a bowl (the spoon was not put into the mouth)
(8) Broom: the participants used a broom as if they cleaned the floor
(9) Match: the participants continuously lit a match using the non-flammable side of the match box
(10) Opening a jar: the participants were handed jars and had to open them using only the instructed hand.

Whenever tasks had to be performed that could not be continuously executed without interruption (e.g., throwing the ball) or required picking up objects (e.g., opening a jar), the experimenter assisted the participant to ensure that only the instructed task was performed. While some tasks required the use of the hand that was not performing the task directly (e.g., holding the paper during cutting with the scissors), participants were instructed to move the other hand as little as possible under such circumstances.

For the WFQ session, the experiment followed the same procedure as for the EHI session. Again, the experiment consisted of 20 trials in total (10 tasks, once per foot) of 1 min duration during which the behavior had to be performed continuously. If the task could not be executed continuously without assistance (e.g., ball kicking), the experimenter assisted by placing, e.g., balls in front of the participant. Individual trials were separated by a 30 s ITI followed by a standardized oral instruction about the next trial. Task order and starting foot were randomized and counterbalanced across participants. The tasks were conducted as follows:

(1) Kicking a ball: the participants continuously kicked balls against the wall. The experimenter placed new balls in front of the instructed foot throughout the trial
(2) Jumping: the participants were asked to jump on the instructed foot for the duration of the trial
(3) Standing: the participants stood one-footed on the instructed foot
(4) Smoothing sand: the participants drew a prepared shape of the number eight with the instructed foot
(5) Stepping onto a chair: the participants continuously stepped onto a small chair (only with the instructed foot)
(6) Weight-shifted standing: the participants stood on the floor putting their weight onto the instructed foot
(7) Shovel: the participants simulated stepping onto a shovel by stepping onto the edges of a large broom
(8) Marble: the participants used their toes to move marbles from one bowl to another
(9) Balancing: the participants stood one-footed on a narrow wooden rail
(10) Bug: the participants continuously stepped onto a dot projected onto the floor by the experimenter.

### EEG Recording, Preprocessing, and Analysis

EEG signals were recorded with a mobile recording system (LiveAmp 32, Brain Products GmbH, Gilching, Germany). The LiveAmp 32 comprises 32 Ag-AgCl electrodes arranged according to the international 10–20 system (C3/C4, FP1/FP2, F3/F4, F7/F8, FC1/FC2, FC5/FC6, FT9/FT10, T7/T8, CP1/CP2, CP5/CP6, TP9/TP10, P3/P4, P7/P8, and O1/O2). The FCz electrode served as primary reference during recording. Signals were amplified using a wireless amplifier (analog-to-digital conversion: 24-bit) and recorded using the Brain Vision analyzer software at a sampling rate of 1000 Hz. Impedances were kept below 10 kHz during the recording session to ensure good signal quality. The EEG system furthermore featured three acceleration sensors in the X, Y, and Z direction located at the backside of the skull that recorded movements of the head.

The signals were preprocessed offline using the *Brain Vision Analyzer* (Brain Products GmbH, Gilching, Germany). Raw data were filtered from 0.1 Hz (high pass filter) to 30 Hz (low pass filter) at a slope of 24 dB/octave. After filtering, all signals were manually inspected to exclude sections containing technical artifacts and to identify channels with poor recording quality. To remove systematic artifacts such as vertical and horizontal eye movements or pulse-related signals, we applied an infomax independent component analysis (ICA) to the remaining data. The FCz channel and channels of poor signal quality were then recalculated via topographic interpolation.

To analyze the data, tasks were first epoched as a whole (60 s duration) and baseline-corrected using a 500 ms window prior to trial onset as the baseline. These large epochs were segmented into 58 non-overlapping segments (1024 ms segment duration). Segments underwent automatic artifact rejection and were excluded if any of the following conditions applied: (1) voltage steps of 50 μV/ms, (2) value differences of more than 200 μV within a 200 ms interval and (3) signal strength below 0.5 μV within a 100 ms interval. We then applied a current source density (CSD; Peters and Servos, 1989) transformation to remove the reference potential from the processed data and finally used a Fast-Fourier transformation to decompose the oscillatory data into different frequency bands (Hammond window of 10%). Only alpha and beta frequencies were analyzed in the context of this study as these have been implicated in motor functioning (Klostermann et al., 2007). Alpha frequencies were defined as frequencies between 8 and 13 Hz and beta frequencies were defined in the range between 13 and 30 Hz. We then averaged the power density (power per unit bandwidth) and extracted it for all tasks individually and for all tasks pooled (all left – condition and all right – condition) per electrode pair (C3/C4, FP1/FP2, F3/F4, F7/F8, FC1/FC2, FC5/FC6, FT9/FT10, T7/T8, CP1/CP2, CP5/CP6, TP9/TP10, P3/P4, P7/P8, and O1/O2). Finally, asymmetry indices (AIs) were computed across the averaged power densities for each individual activity and for all left and all right tasks combined using the formula: AI = (power right – power left)/(power right + power left). The processing steps were identical for both the EHI session and the WFQ session.

### Statistical Analysis

Statistical analyses were performed using SPSS (version 21, Chicago, IL, United States). For the alpha band analysis of the EHI session, asymmetric electrode sites were identified across all task conditions by applying a repeated-measures ANCOVA with the within-subjects factors side of task performance (left or right) and electrode pair as well as the between-subjects factor handedness. We used the X, Y, and Z acceleration sensor signals during the task segments as covariates to identify whether head movements during these behaviors had a significant influence on task-related variables in the recordings. Here, we used the raw signal of the accelerometers as covariate as we wanted to identify how actual rather than processed movement signals influence the physiological signal. Focusing on the most significantly asymmetric electrode pair, we conducted a 10 × 2 × 2 ANCOVA with all individual tasks of the questionnaire and side of the task performance as within-subjects factor and handedness as between-subjects factor. Again, X, Y, and Z acceleration sensor signals were used as covariates. Finally, we used multiple linear regression with all left-sided tasks as predictors or all right-sided tasks as predictors to identify whether the neuronal signal could predict the EHI score of participants. Here, we repeated the analysis steps conducted for the EHI session. For the WFQ session, we used footedness as a between-subjects variable rather than handedness (identical cut-offs). These measures were strongly correlated indicating that lateral biases in handedness and footedness in individuals within our sample were associated [two-sided Pearson correlation: *r*(50) = 0.689, *p* < 0.001]. However, even though mixed-handers were excluded, a large fraction of participants (*n* = 19) exhibited mixed-footedness. The footedness analysis was therefore conducted in two steps. First, analogous to the handedness analysis, only left- and right-footed participants were compared. In a second step, participants with mixed foot preference were included into the analysis to identify whether they exhibited neurophysiological activity different from both left- and right-footed participants. All aforementioned analyses were conducted identically for alpha and beta frequency bands.

## Results

### EHI

#### EEG Alpha Asymmetries

**FIGURE 1:**
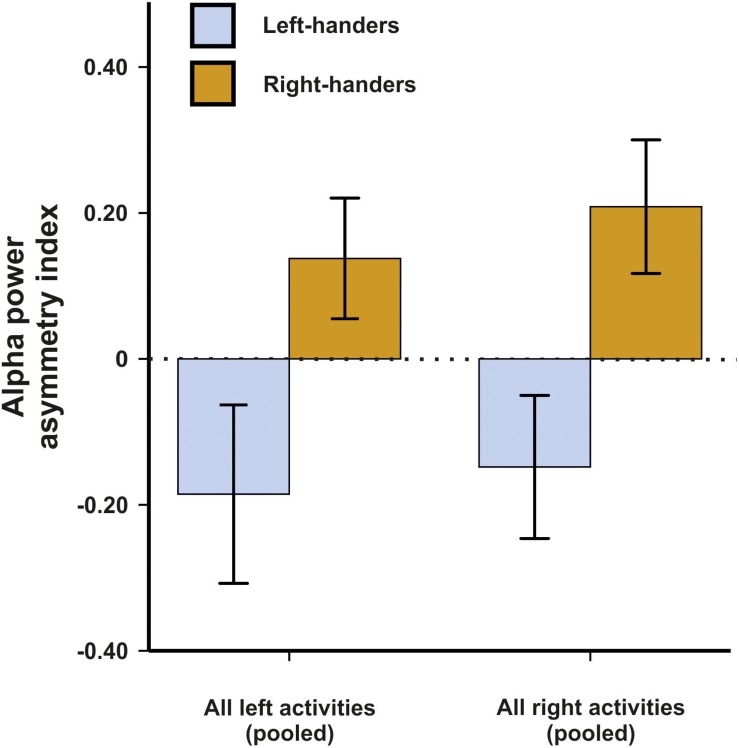
Alpha power asymmetries between the left and right hemisphere during left and right task performance at the FC5/FC6 electrode pair. Error bars represent ± 1 SEM.

In a first analysis, we aimed to determine which electrode pair differentiated best between left- and right-handers in these motor tasks. While we expected that motor asymmetries demonstrate the strongest effects at fronto-central sites roughly overlapping with motor cortex or premotor cortex (Schmitz et al., 2019), we chose to use a non-biased approach due to the novelty of the used EEG system. We therefore performed a 14 × 2 × 2 ANCOVA with the within-subjects factors electrode pair (C3/C4, FP1/FP2, F3/F4, F7/F8, FC1/FC2, FC5/FC6, FT9/FT10, T7/T8, CP1/CP2, CP5/CP6, TP9/TP10, P3/P4, P7/P8, and O1/O2) and side of task performance (left and right) as well as the between-subjects factor handedness (left-handers and right-handers). Average movements in the X, Y and Z direction were included as covariates in the ANCOVA model to correct for potential movement effects. There was no main effect of electrode pair, side of task performance or handedness (all *F*s < 1.44). The interaction between the factor electrode pair and handedness however reached significance in the ANCOVA indicating that some electrode pairs differentiated better between left- and right-handers than others [*F*(9,414) = 2.27, *p* = 0.006, η^2^ = 0.05]. Bonferroni-corrected *post hoc* tests revealed that the best differentiation between left- and right-handers was found on electrodes FC5 and FC6 (*p* = 0.011). The FC electrodes correspond to supplementary motor and premotor areas (Puzzo et al., 2010). Here, left-handers had a negative asymmetry index indicating greater alpha power in the left hemisphere whereas right-handers had a positive asymmetry index indicating greater alpha power in the right hemisphere (Figure 1). We therefore decided to focus on this electrode pair throughout the manuscript in all further analyses. Importantly, neither the X, Y, or Z direction for movement signals exhibited a significant influence indicating that the behavioral tasks were unaffected by movement artifacts (all *p*s > 0.250). Thus, despite considerable movement during the experiment, they were no systematic effects on hemispheric asymmetries, likely because they are a relative measure and movement signals are found equally in both hemispheres.

**FIGURE 2:**
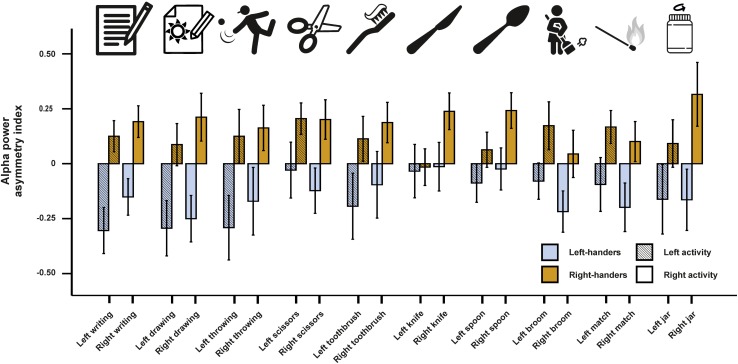
Alpha power asymmetries between the left and right hemisphere at the FC5/FC6 electrode pair for all individual tasks of the Edinburgh Handedness Inventory (EHI). Tasks from left to right: writing the alphabet, drawing a sketch, throwing a ball, cutting a shape with scissors, brushing teeth, cutting clay with a knife, eating soup with a spoon, cleaning the floor with a broom, striking a match and opening jars. Error bars represent ± 1 SEM.

In a next step, we investigated alpha power asymmetries for all individual tasks at the FC5/FC6 electrode site. We computed a 10 × 2 × 2 ANCOVA with the 10 tasks (writing, throwing a ball, drawing, using scissors, brushing teeth, using a knife, using a spoon, using a broom, striking a match and opening a box-lid/jar) and side of the task performance (left and right) as within-subject factors. Handedness was again used as a between-subjects factor and the X, Y, and Z direction movement accelerators were included as covariates. We found a significant main effect of handedness [*F*(1,46) = 7.55, *p* = 0.009, η^2^ = 0.14]. There were no significant interactions with handedness or any of the movement parameters (*p*s > 0.087). Power spectra for the movement accelerometers of each individual task are depicted in Supplementary Figure S1. Alpha power asymmetries for all individual tasks are depicted in Figure 2.

Finally, we used multiple linear regression to identify whether tasks performed on the left and tasks performed on the right side could significantly predict the LQ of the participants. Using left-sided tasks as predictors, the model reached significance [*F*(10,40) = 2.40, *p* = 0.024, adjusted *R*^2^ = 0.22]. Here, the only individual predictor reaching significance was writing (*p* = 0.006). For right-sided tasks, the model did not reach significance [*F*(10,40) = 1.03, *p* > 0.250, *R*^2^ = 0.05].

#### EEG Beta Asymmetries

**FIGURE 3:**
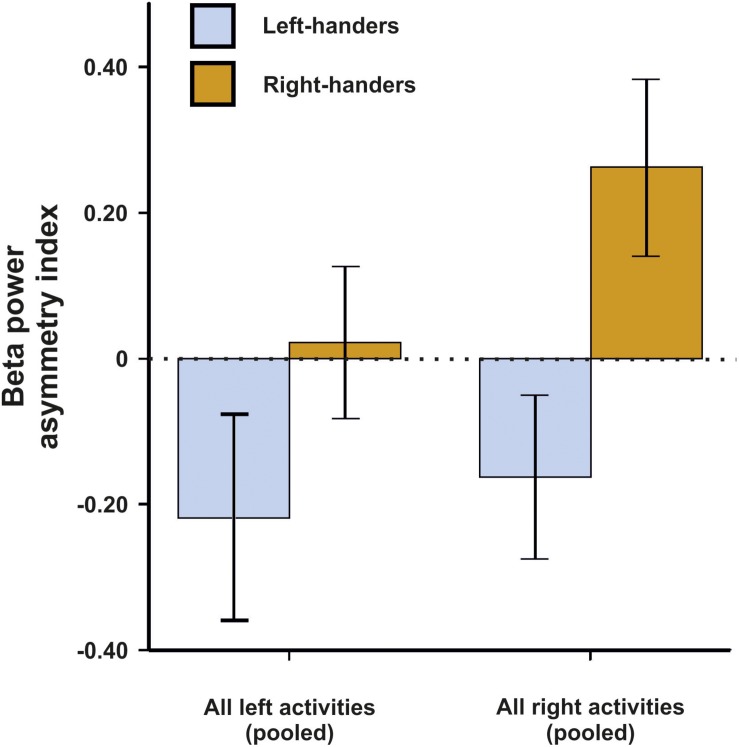
Beta power asymmetry for both left- and right-handers during left- and right-sided tasks (pooled) of the EHI. Error bars represent ± 1 SEM.

**FIGURE 4:**
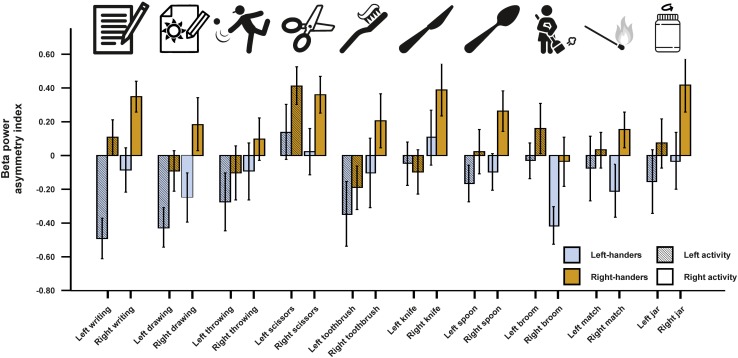
Beta power asymmetry for all individual tasks of the EHI for left- and right-handers during left- and right-sided performance. Error bars represent ± 1 SEM.

For beta asymmetries, we repeated the analysis as for alpha asymmetries (10 × 2 × 2 ANCOVA with task and side of task performance as within-subjects factors and handedness as between-subjects factor). Again, we first checked whether movement artifacts as measured by the X, Y, and Z acceleration sensors had a significant influence on the signal. There were no significant results for any direction (all *p*s > 0.250). We could not detect any significant main effects of task, side of task performance or handedness (all *p*s > 0.135). However, there was a significant interaction between the side of the task performance and handedness [*F*(1,45) = 4.33, *p* = 0.043, η^2^ = 0.09]. Bonferroni-corrected *post hoc* tests revealed that beta power asymmetries were significantly different between left- and right-sided tasks for both left- and right-handers (*p* = 0.033 and *p* < 0.001, respectively). The results for all tasks combined are depicted in Figure 3. The results for all individual tasks are shown in Figure 4.

Using the beta asymmetry indexes from left-sided tasks as predictors for the EHI score in a multiple linear regression model demonstrated a trend [*F*(10,40) = 2.01, *p* = 0.058, adjusted *R*^2^ = 0.17]. Here, as for alpha asymmetries, the only significant predictor was the writing item of the EHI (*p* = 0.002). For right-sided tasks, the model was not significant [*F*(10,40) = 1.15, *p* > 0.250, adjusted *R*^2^ = 0.03].

### WFQ

#### EEG Alpha Asymmetries

**FIGURE 5:**
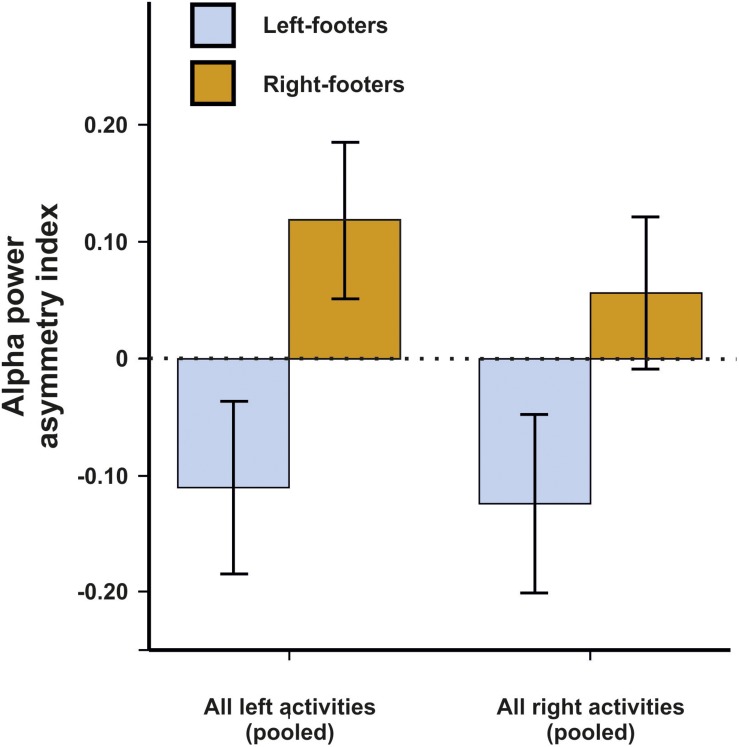
Alpha power asymmetries between left- and right-footers at the FC5/FC6 electrode site for all pooled tasks of the WFQ. Error bars represent ± 1 SEM.

**FIGURE 6:**
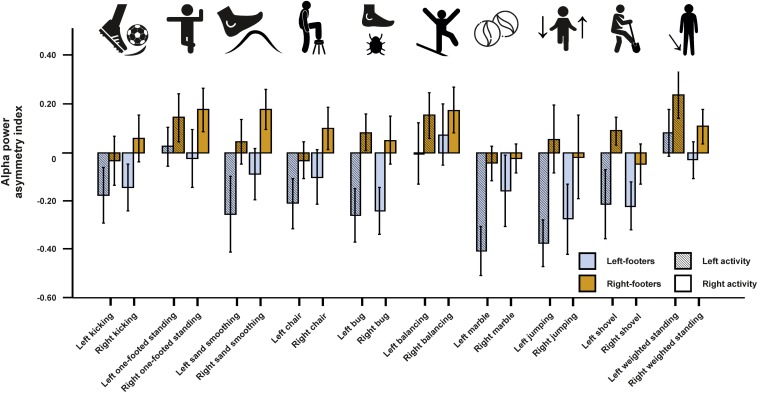
Alpha power asymmetries for all individual tasks of the WFQ for left- and right-footers during left and right task performance. Individual tasks from left to right: kicking a ball, standing on one foot, simulated sand-smoothing, stepping onto a chair, stepping onto a bug (laser dot), balancing on a rail, grasping a marble, jumping one-footed, simulated step on a shovel (broom), weight-shifted relaxed standing. Error bars represent ± 1 SEM.

We first conducted a repeated-measures ANCOVA for the alpha power asymmetries using all left- and all right-sided tasks pooled as within-subjects variable and footedness as between-subjects variable. In a first step, only left- and right-footers were analyzed. We found that the FC5/FC6 electrode sites differentiated significantly between left- and right footers [*F*(1,26) = 4.45, *p* = 0.045, η^2^ = 0.15, Figure 5]. However, there was no interaction between footedness and the side of the task performance (*F* < 1), nor with any of the acceleration sensors. We then conducted a 10 × 2 × 2 ANCOVA with the WFQ tasks (ball kicking, hopping on one leg, standing on one foot, smoothing sand, stepping onto a chair, weight-shifted relaxed standing, stepping on a shovel, grasping a marble, balancing on a rail and stepping on a bug) and side of task performance (left and right) as within-subjects variables and footedness as between-subjects variable. The ANCOVA for alpha power asymmetries demonstrated a significant main effect of footedness [*F*(1,26) = 6.05, *p* = 0.021, η^2^ = 0.20]. No interaction with task, task side or any movement parameter reached significance (all *p*s > 0.064, Figure 6). Power spectra for the three movement accelerometers can be found in Supplementary Figure S2.

As before, multiple linear regression analysis was used to identify if the alpha power asymmetries in left- or in right-sided tasks could significantly predict the WFQ score. The model reached significance for left-sided tasks [*F*(10,29) = 2.52, *p* = 0.040, adjusted *R*^2^ = 0.343], but not for right-sided tasks (*F* < 1). For left-sided tasks, only the beta-weight for grasping a marble was significant (*p* = 0.006).

Since a considerable number of our participants exhibited mixed-footedness (*n* = 19), we repeated the analysis including mixed-footers. There was again a main effect of footedness [*F*(2,44) = 3.36, *p* = 0.044, η^2^ = 0.13]. *Post hoc* test revealed a significant difference between left- and mixed-footers in their alpha asymmetry levels (*p* = 0.041) whereas the difference between left- and right-footers did not reach significance (*p* = 0.208, see Supplementary Figure S3). No other main effect or interaction reached significance in this analysis (all *F*s < 1.06). The 10 (task) × 2 (side) × 3 (footedness) ANCOVA demonstrated a significant main effect of footedness [*F*(2,42) = 3.64, *p* = 0.035, η^2^ = 0.15, see Supplementary Figure S4]. Again, left-footers had significantly lower AIs compared to mixed-footers (*p* = 0.035), but not compared to right-footers (*p* = 0.150).

Both left- and right-sided task activity failed to significantly predict the WFQ score when mixed-footers were included in the analysis [*F*(10,37) = 1.11, *p* = 0.382, adjusted *R*^2^ = 0.022 for left-sided tasks; *F*(10,37) = 0.72, *p* = 0.699, adjusted *R*^2^ = −0.06 for right-sided tasks]. However, hopping on the left leg could significantly predict the WFQ score (*p* = 0.029) whereas no right-sided activity could predict WFQ scores.

#### EEG Beta Asymmetries

**FIGURE 7:**
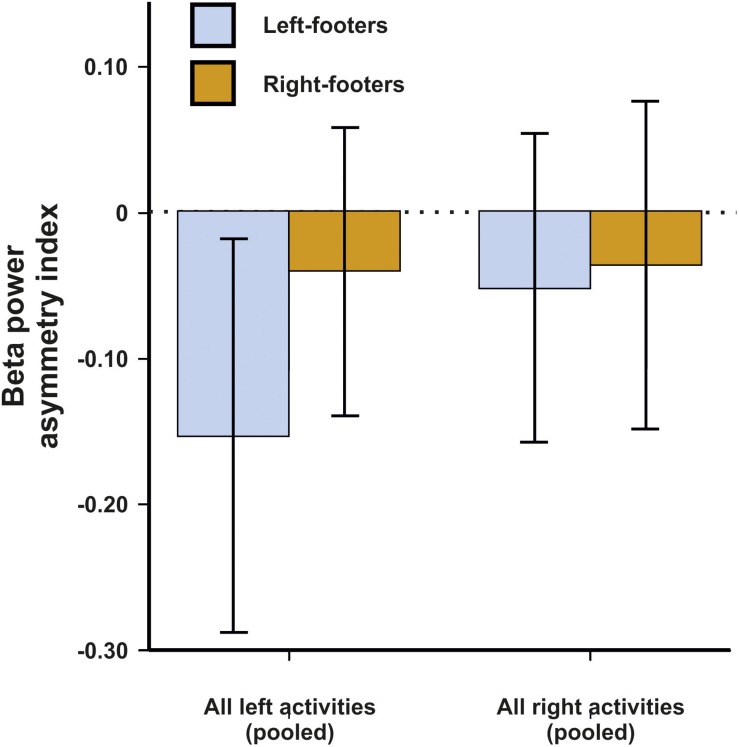
Beta power asymmetries for all pooled tasks of the WFQ for left- and right-footers during left and right task performance. Error bars represent ± 1 SEM.

**FIGURE 8:**
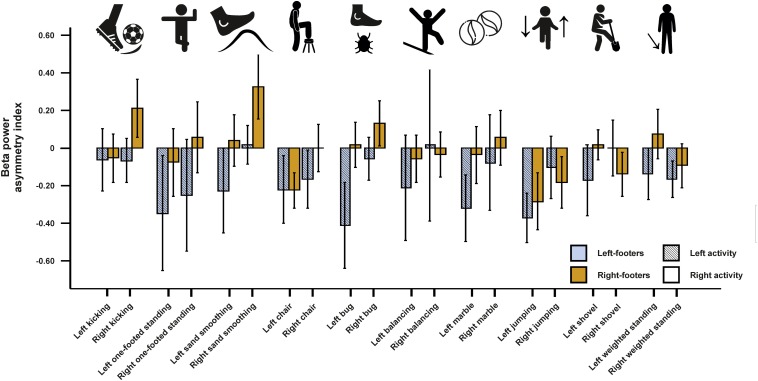
Beta power asymmetries for all individual tasks of the WFQ for left and right-footers during left and right task performance. Error bars represent ± 1 SEM.

For beta asymmetries, the repeated-measures ANCOVA using all left- and all right-sided tasks pooled as within-subjects variable and footedness as between-subjects variable did not demonstrate any significant results (Figure 7). The same was true for the ANCOVA including all individual tasks (Figure 8) and the multiple linear regression for left- and right-sided task performance.

The inclusion of mixed-footers in the analysis did not change the result pattern as neither the ANCOVA for all left-sided and right-sided tasks (see Supplementary Figure S5), the ANCOVA including all individual items (see Supplementary Figure S6), nor the multiple linear regression for left- and right-sided tasks reached significance.

## Discussion

In the present study, we investigated the neurophysiological correlates of the most prominent handedness and footedness questionnaires—the EHI and the WFQ—in a real-life setting with high ecological validity. A mobile EEG system was used that allows for neural recording during active movement. We hypothesized that alpha and beta power asymmetries could differentiate both between left- and right-handers and between left- and right-footers during the performance of EHI and WFQ tasks. Furthermore, we hypothesized that these signals can distinguish between tasks performed on the left or on the right. Our results show that alpha power asymmetries at the FC5/FC6 electrode sites distinguished significantly between left- and right-handers for EHI items. The same was true for the distinction between left- and right-footers based on alpha power asymmetries during the performance of WFQ items. While we found no main effect of task side, we found significant interactions between task side and handedness in the beta frequency band for EHI items. These results were unaffected by movement artifacts since no analysis was significantly influenced by movement parameters as measured by the acceleration sensors. Finally, we could predict both the EHI and WFQ score based on alpha power asymmetries during tasks performed on the left side, but not on the right side using multiple linear regression analysis.

### Edinburgh Handedness Inventory

For the EHI, results for the pooled tasks of all EHI items showed a significant difference between left- and right-handers in the alpha frequency band. Here, left-handers had negative alpha asymmetry power and right-handers had positive alpha power asymmetries. Oscillatory alpha activity has been associated with functional inhibition which has been demonstrated in visuospatial attention tasks (Worden et al., 2000; van der Werf et al., 2008; Kelly et al., 2009), but also during facial recognition (Jokisch and Jensen, 2007) and somatosensory working memory tasks (Haegens et al., 2010). This functional inhibition is hypothesized to be generated by rhythmic GABAergic input from local interneurons (Jensen and Mazaheri, 2010). Thus, the detected alpha power asymmetries in our study indicate that there was a stronger activation of the right hemisphere in left-handers (stronger left-hemispheric alpha power - > stronger left-hemispheric inhibition) and a stronger activation of the left hemisphere in right-handers (stronger right-hemispheric alpha power - > stronger right-hemispheric inhibition). These activation patterns are well in line with findings from fMRI and TMS studies demonstrating that movements of the dominant hand are generally associated with a stronger activation of the contralateral hemisphere (van den Berg et al., 2011; Grabowska et al., 2012). However, these studies also found that voluntary movements with the non-dominant hand are associated with more bilateral activation patterns indicating that the contralateral hemisphere to the dominant hand is generally implicated in the control of movement, regardless of the movement’s body side. This result could explain why we found no significant interaction between the side of the task performance and handedness.

On the physiological level, we found no effects of individual tasks and their interactions with handedness. This finding contrasts studies investigating the factorial validity of the EHI questionnaire, which display that some items such as the usage of a broom or opening a box/jar load poorly on a single handedness factor (Dragovic, 2004; Milenkovic and Dragovic, 2013) probably due to the necessity of using the other hand as well for these tasks. A possible explanation for the discrepancy between behavioral and neurophysiological findings in our study might be attributed to a lack of power since item differences, if existing, are seemingly small in effect and therefore not easily detected by classical hypothesis testing.

While we did not find any differences between the items in the ANCOVA, we could find a significant prediction of the writing item in left-sided movements for the LQ in the alpha frequency band indicating that this item provides the best estimate for handedness on the neurophysiological level. A corresponding finding was not evident for right-sided movements where no item could significantly predict the EHI score. A possible reason for the discrepancy between the predictive value of left vs. right task performance side can be found in differences in bilateral hand skill between left- and right-handers. Left-handers outperform right-handers in tasks involving a coordination between the left and the right hand (Judge and Stirling, 2003), perform almost equally well in fine-tuned motor task with the left and the right hand (Schmitz et al., 2019) and are better in faking right-handedness than right-handers faking left-handedness (McManus et al., 2018). Right-sided tasks are thus inadequate to differentiate between left- and right-handers as left-handers are usually very skilled in using their right hand. Therefore, neural activation patterns during tasks performed with the right hand are unlikely to provide substantial predictive value for the EHI score.

For the beta frequency band, we found comparable results to the alpha frequency analysis. Although an increase in beta power is not directly associated with functional inhibition, voluntary movements have been linked to decreases in beta power (Hammond et al., 2007). Therefore, our finding of higher beta power in the hemisphere that is not dominantly controlling the execution of the movement (i.e., higher beta power in the right hemisphere for right-sided movements and higher beta power in the left hemisphere for left-sided movements) fits into this framework. It has to be noted however that this hypothesis has been challenged and that the association between motor function and beta power still remains unclear (Jenkinson and Brown, 2011). Furthermore, a recent study of Ocklenburg et al. (2019) found strong correlations between asymmetries in the alpha and beta frequency band indicating that alpha and beta frequency bands might have functionally similar roles.

Notably, the interaction between task performance side and handedness was stronger in right-handers than left-handers, again indicating that right-handers are more lateralized compared to left-handers during motor tasks on the neural level. However, this finding could also be a result of our chosen cut-offs since the classification for left- and right-handers was symmetrical around 0 (LQ < −40 = left-handed, LQ > 40 = right-handed). A large-scale psychometric study has however demonstrated that cut-offs for left- and right-handers are not symmetrical around 0 for a refined 10-item scale based on the EHI (LQ < −7 = left-handed, LQ > 72 = right-handed; Tran et al., 2014). Unfortunately, the exclusion of mixed-handers from the analysis does not allow for definite conclusions on this matter as using the asymmetrical cut-offs as defined by Tran et al. (2014) only marginally changes the classification for left- and right-handers in our study. Future studies should therefore include mixed-handers to identify whether symmetrical or asymmetrical cut-offs significantly alter the neurophysiological result patterns as hinted at by extensive psychometric testing on the behavioral level (Tran et al., 2014). As for alpha asymmetries, no interaction between individual tasks of the EHI and handedness could be found indicating that there were no major differences between the items. However, multiple linear regression analysis again revealed a significant beta-weight for left-sided writing indicating that writing provides the best fit when estimating handedness based on neurophysiological activity.

### Waterloo Footedness Questionnaire

For the WFQ session, we only found significant effects of footedness in the alpha frequency band indicating that items of the WFQ can differentiate between left- and right-footers on the neural level. As for handedness, there were no interactions with the side of the task performance. This lack of an interaction could again be attributed to bilateral activation patterns regardless of the side of execution. In fMRI studies, foot movements have been shown to be less lateralized than hand or finger movements (Kapreli et al., 2006; Rocca and Filippi, 2010). However, as for handedness, movement execution with the non-dominant foot displayed more bilateral activation patterns as compared to movements performed with the dominant foot (Rocca and Filippi, 2010). Comparable to the EHI, we found no interaction effects of individual tasks with footedness in the ANCOVA. However, multiple linear regression analysis indicated that grasping a marble is predictive of the participants’ WFQ score, possibly due to the fine-tuned movement necessary in this condition. Since there has been no study investigating the factorial validity of the WFQ, this study provides a first insight into item-specific differentiation between left- and right-footers. Interestingly, the classically used item of kicking a ball did not reach significance here, possibly due to the necessity of stabilizing the body with the non-kicking foot resulting in bilateral activation patterns both in left- and right-footers.

### Limitations

A shortcoming of the present study concerns the rather small sample size of the study. Low sample sizes heighten the probability for false negative results meaning that existing effects could not be detected due to low power (Button et al., 2013). Especially the non-significant differences between individual items both for the EHI and the WFQ could be attributed to a lack of power rather than a lack of differentiation between the items. Brysbaert (2019) noted that small effects can only be reliably detected if individual groups consist of at least 100 participants. Thus, our study only had the possibility to reveal medium to large effects, a problem that was increased in our footedness analysis due to the even smaller sample sizes per individual group. We want to stress however that the main effects between left- and right-lateralized participants were large indicating that the sample size was adequate to demonstrate the viability of mobile EEGs in neuroscientific research in general. Since these results are conform to a wide body of literature on motor asymmetries, we are confident in their validity. Nonetheless, it has to be noted that for example a large-scale imaging study on cortical asymmetry could for example not replicate effects found in previous studies with smaller sample sizes (Guadalupe et al., 2014). Therefore, the results of the present study need to be replicated and extended in larger datasets to ensure the validity of our findings and to clarify whether the non-significant difference between items was a result of a lack in statistical power.

Another limitation of the study can be found in the *a priori* exclusion of mixed-handers as this group might have been informative regarding the specificity of our findings for left- and right-handers. We chose to exclude this group from the current study as we found very few mixed-handers according to the cut-offs used in this study during the pre-screening process. However, there was a considerable fraction of mixed-footers in the dataset that allowed for a comparison between left-, mixed-, and right-footers. We found that mixed-footers resemble right-footers in their neural activity patterns, a results that might have been due to the majority of mixed-footers being lateralized to the right rather than to the left (*n* = 7 for LQ < 0, *n* = 12 for LQ > 0). Interestingly, the mixed-footers seemed to be even stronger lateralized compared to right-handers. However, this result might have been due to variance in the signal and the reduced sample sizes per group.

Finally, it should be noted that we identified the electrode pair of interest by using the behavioral assessment of handedness. This induces a certain circularity into the analysis as we try to distinguish between left- and right-handers on the physiological level, but used the EHI score as a tool to find the strongest distinction between them. Since the multiple linear regression analyses indicate that the physiological signal can predict the EHI score (at least for individual items), a data driven approach to identify the handedness of the participants should however be possible using EEG. Future studies could use, e.g., machine learning tools to completely circumvent a behavioral assessment and try to identify handedness merely by the physiological activity.

### Future Directions

This study is the first to use a mobile EEG system to investigate motor laterality during real-life motor behavior. Since we could replicate known result patterns from fMRI studies on motor laterality, this methodology seems to work appropriately. Therefore, it could be applied to other measures for handedness and footedness other than the EHI and the WFQ as recent studies have come up with new and more refined scales for both phenotypes (Tran et al., 2014; Tran and Voracek, 2016). Especially for footedness, Tran and Voracek (2016) could show that it is two-dimensional rather than a one-dimensional measure which could be reflected in different neurophysiological patterns in relation to the dimension of the specific items.

Furthermore, the mobile EEG could be used for studying motor laterality other than handedness and footedness. To fully understand the neural mechanisms of lateralized behavior, a large variety of lateralized phenotypes should be investigated. One recently highlighted field of research that could be studied using this novel approach are social touch behaviors. Prominent social touch phenomena such as embracing, kissing, and cradling have been shown to be lateralized on the population level (Ocklenburg et al., 2018). Several studies by our group have found that these biases are both determined by motor biases such as handedness or footedness, but also by the emotional context of the situation (Packheiser et al., 2019a, b). However, since all these studies used purely behavioral approaches, the underlying neurophysiology of social touch remains largely unknown. Mobile EEG as used in the present study could provide a powerful tool to study behavior in more ecologically valid real-life situations and thus could illuminate this field of research.

## Data Availability Statement

The datasets generated for this study are available on request to the corresponding author.

## Ethics Statement

The studies involving human participants were reviewed and approved by Local ethics committee of the Faculty of Psychology, Ruhr-University Bochum. The patients/participants provided their written informed consent to participate in this study.

## Author Contributions

JP and JS conceived and supervised the experiment, analyzed the data, and wrote the manuscript. YP collected the data and reviewed the manuscript. YE and PF helped with data acquisition and data analysis and reviewed the manuscript. OG acquired funding and reviewed the manuscript. SO conceived the experiment, analyzed the data, acquired funding and reviewed the manuscript.

## Conflict of Interest

The authors declare that the research was conducted in the absence of any commercial or financial relationships that could be construed as a potential conflict of interest.

## Supplementary Material

FIGURE S1 Power spectra for the **(A)** X-, **(B)** Y-, and **(C)** Z-head movement direction as measured by the accelerometers for the EHI tasks. Note that all individual activities demonstrate high delta power (oscillations between 0 and 4 Hz). All other frequency bands are almost absent in the signals. Movement in the Y-direction, i.e., back and forth movement of the head, exhibited the strongest amplitudes across all tasks.

FIGURE S2 Power spectra for the **(A)** X-, **(B)** Y-, and **(C)** Z-head movement direction as measured by the accelerometers for the WFQ tasks. As for EHI tasks, delta power dominated the accelerometer signal strength in all individual tasks. Only jumping on one leg induced noticeable amplitudes in faster oscillating frequency bands.

FIGURE S3 Alpha power asymmetries between left-, mixed-, and right-footers at the FC5/FC6 electrode site for all pooled tasks of the WFQ. Error bars represent ± 1 SEM.

FIGURE S4 Alpha power asymmetries for all individual tasks of the WFQ for left-, mixed-, and right-footers during left and right task performance. Error bars represent ± 1 SEM.

FIGURE S5 Beta power asymmetries for all individual tasks of the WFQ for left-, mixed-, and right-footers during left and right task performance. Error bars represent ± 1 SEM.

FIGURE S6 Beta power asymmetries for all individual tasks of the WFQ for left-, mixed-, and right-footers during left and right task performance. Error bars represent ± 1 SEM.
