# Supplementary figures and images for: Using Mobile EEG to Investigate Alpha and Beta Asymmetries During Hand and Foot Use
Source: Front Neurosci. 2020 Feb 14;14:109. doi: 10.3389/fnins.2020.00109 (PMC7033815; doi:10.3389/fnins.2020.00109)

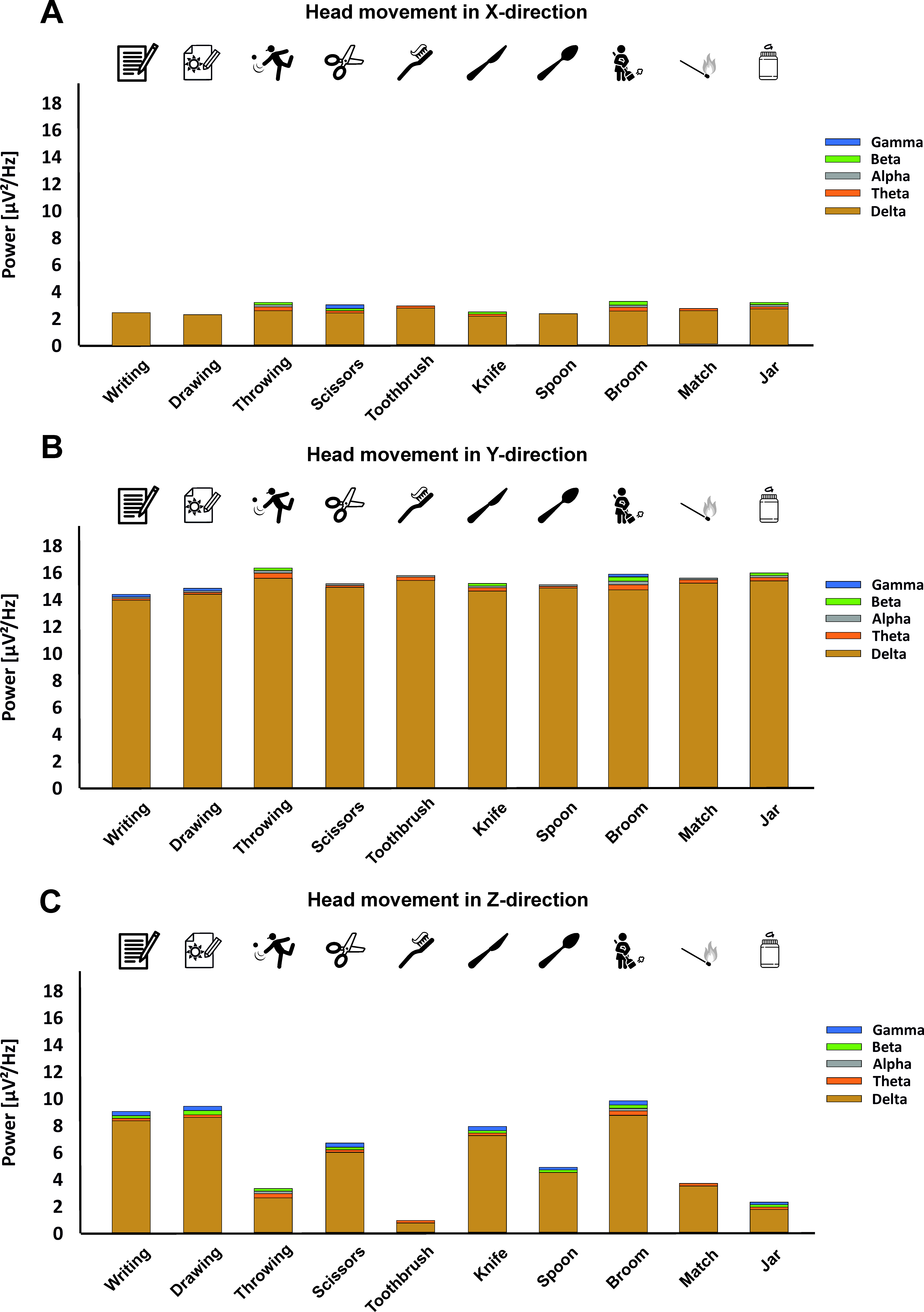

Supplement: FIGURE S1 — Power spectra for the (A) X-, (B) Y-, and (C) Z-head movement direction as measured by the accelerometers for the EHI tasks. Note that all individual activities demonstrate high delta power (oscillations between 0 and 4 Hz). All other frequency bands are almost absent in the signals. Movement in the Y-direction, i.e., back and forth movement of the head, exhibited the strongest amplitudes across all tasks. [file Image_1.jpeg]

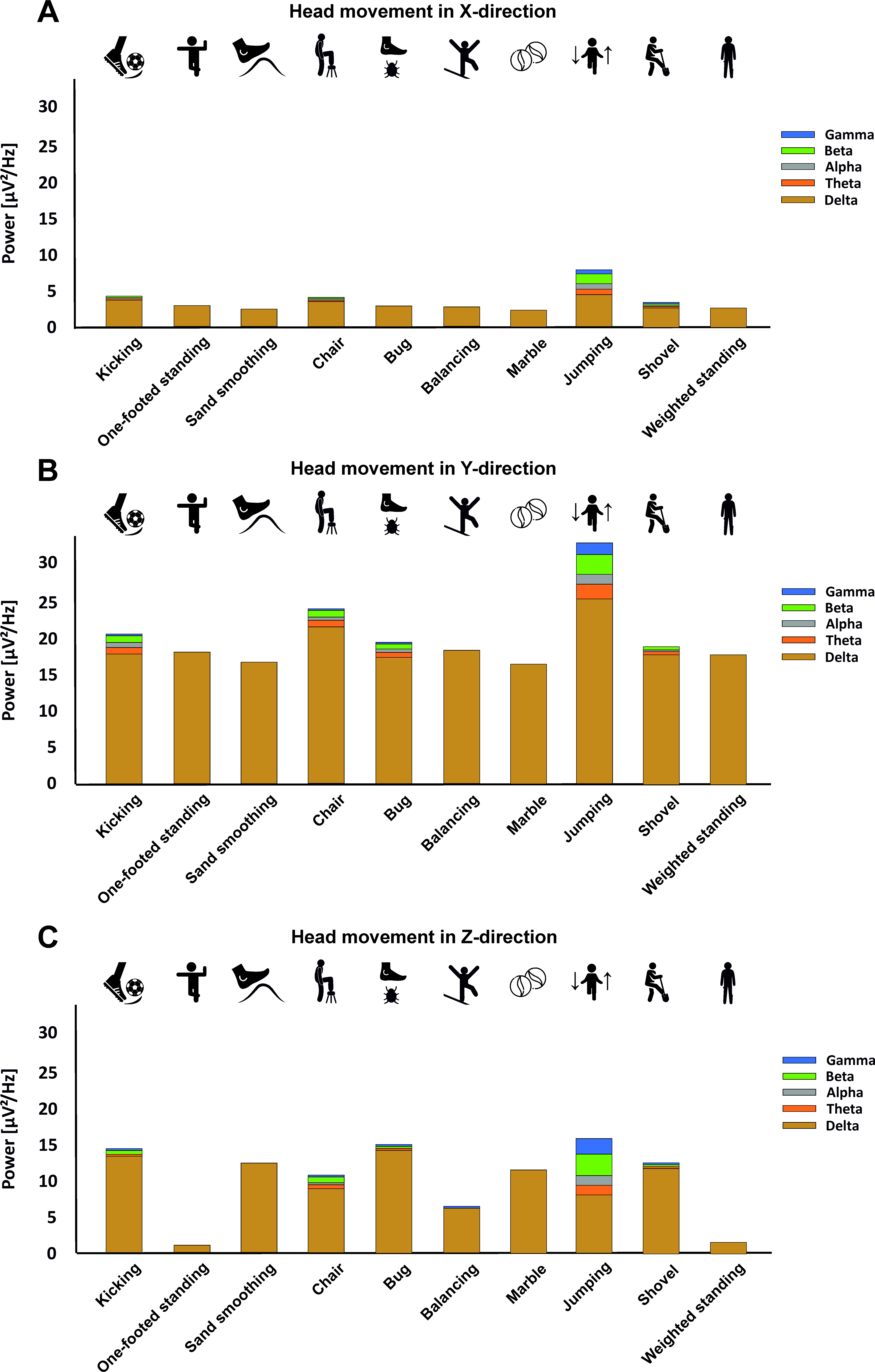

Supplement: FIGURE S2 — Power spectra for the (A) X-, (B) Y-, and (C) Z-head movement direction as measured by the accelerometers for the WFQ tasks. As for EHI tasks, delta power dominated the accelerometer signal strength in all individual tasks. Only jumping on one leg induced noticeable amplitudes in faster oscillating frequency bands. [file Image_2.jpeg]

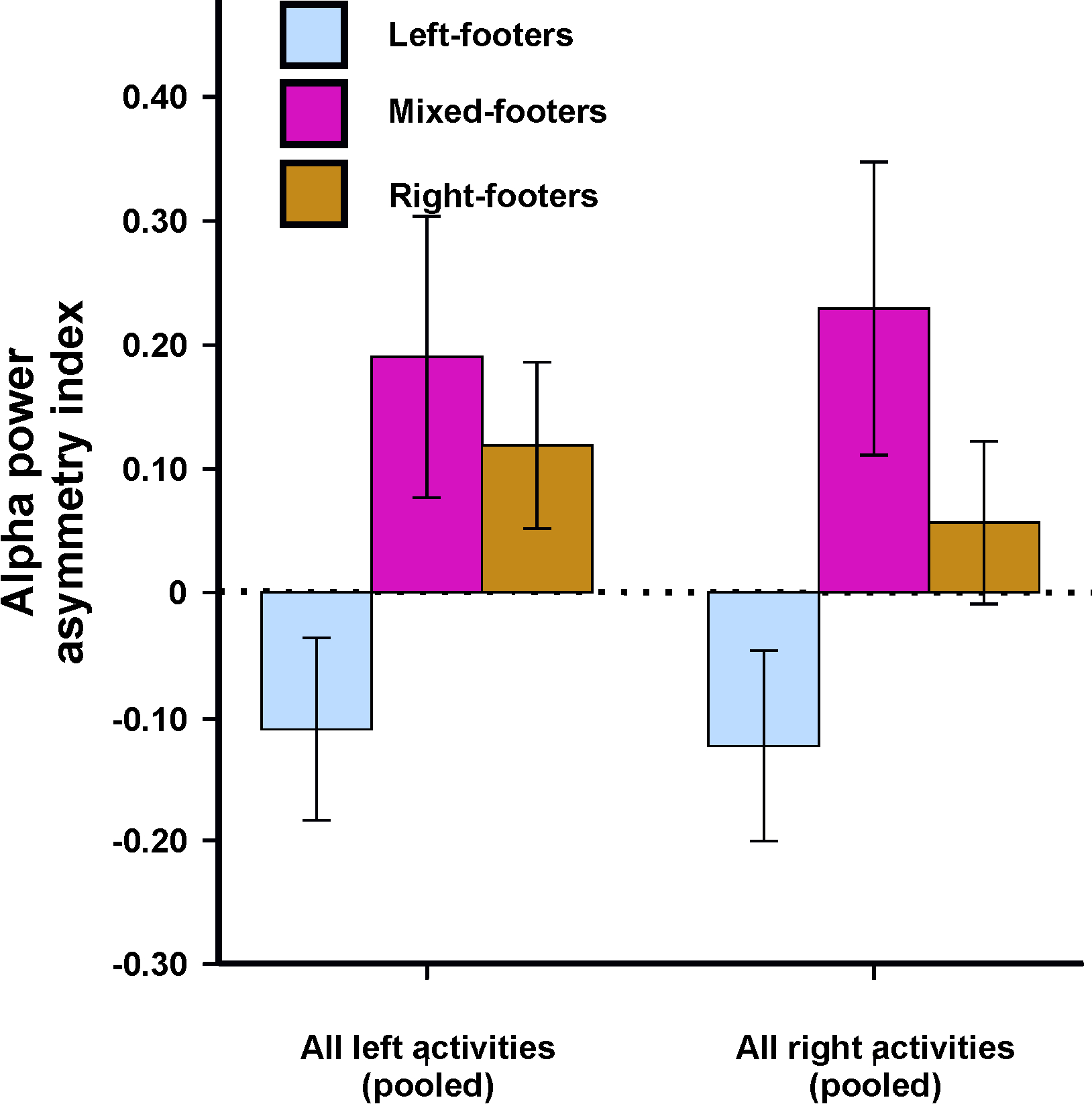

Supplement: FIGURE S3 — Alpha power asymmetries between left-, mixed-, and right-footers at the FC5/FC6 electrode site for all pooled tasks of the WFQ. Error bars represent ± 1 SEM. [file Image_3.jpg]

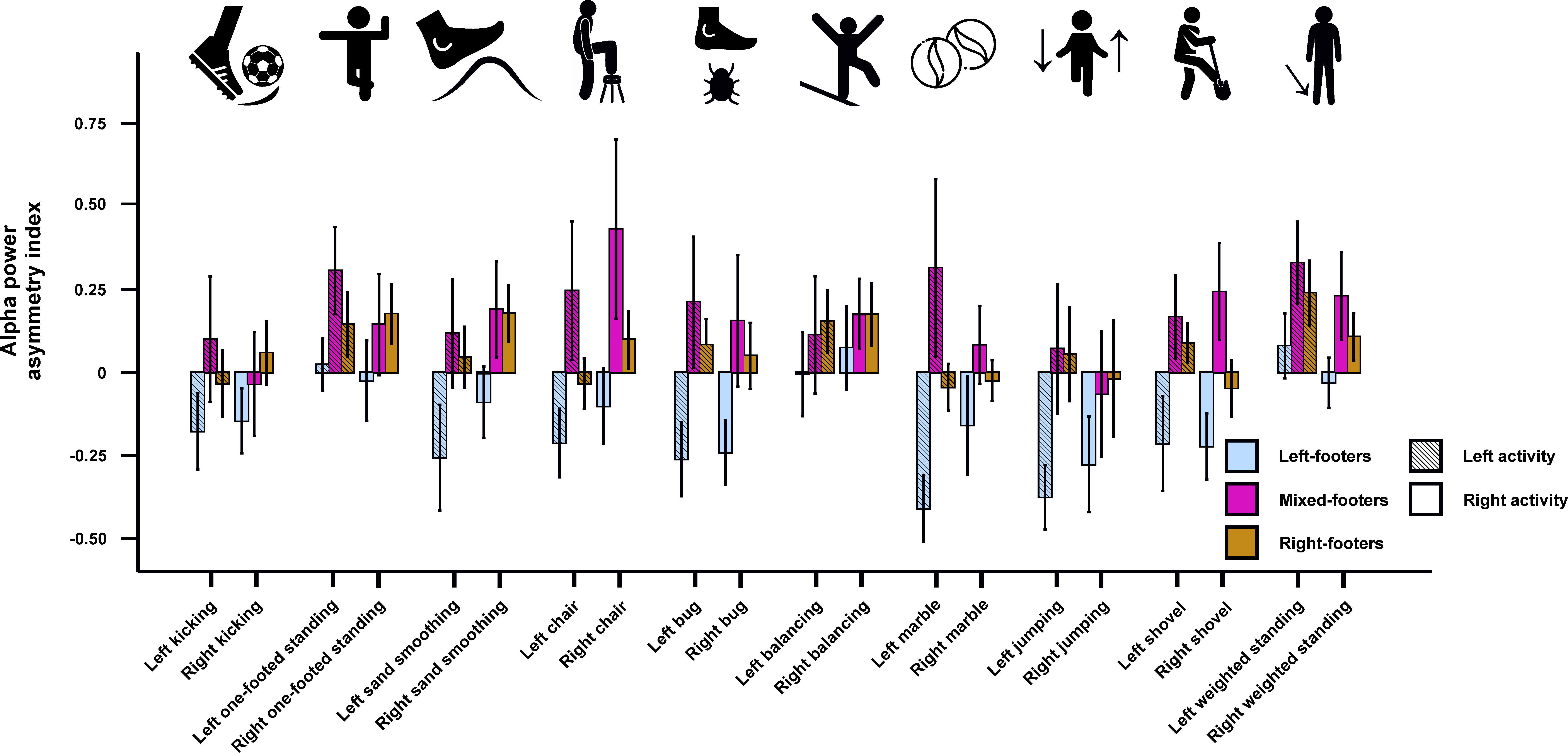

Supplement: FIGURE S4 — Alpha power asymmetries for all individual tasks of the WFQ for left-, mixed-, and right-footers during left and right task performance. Error bars represent ± 1 SEM. [file Image_4.jpeg]

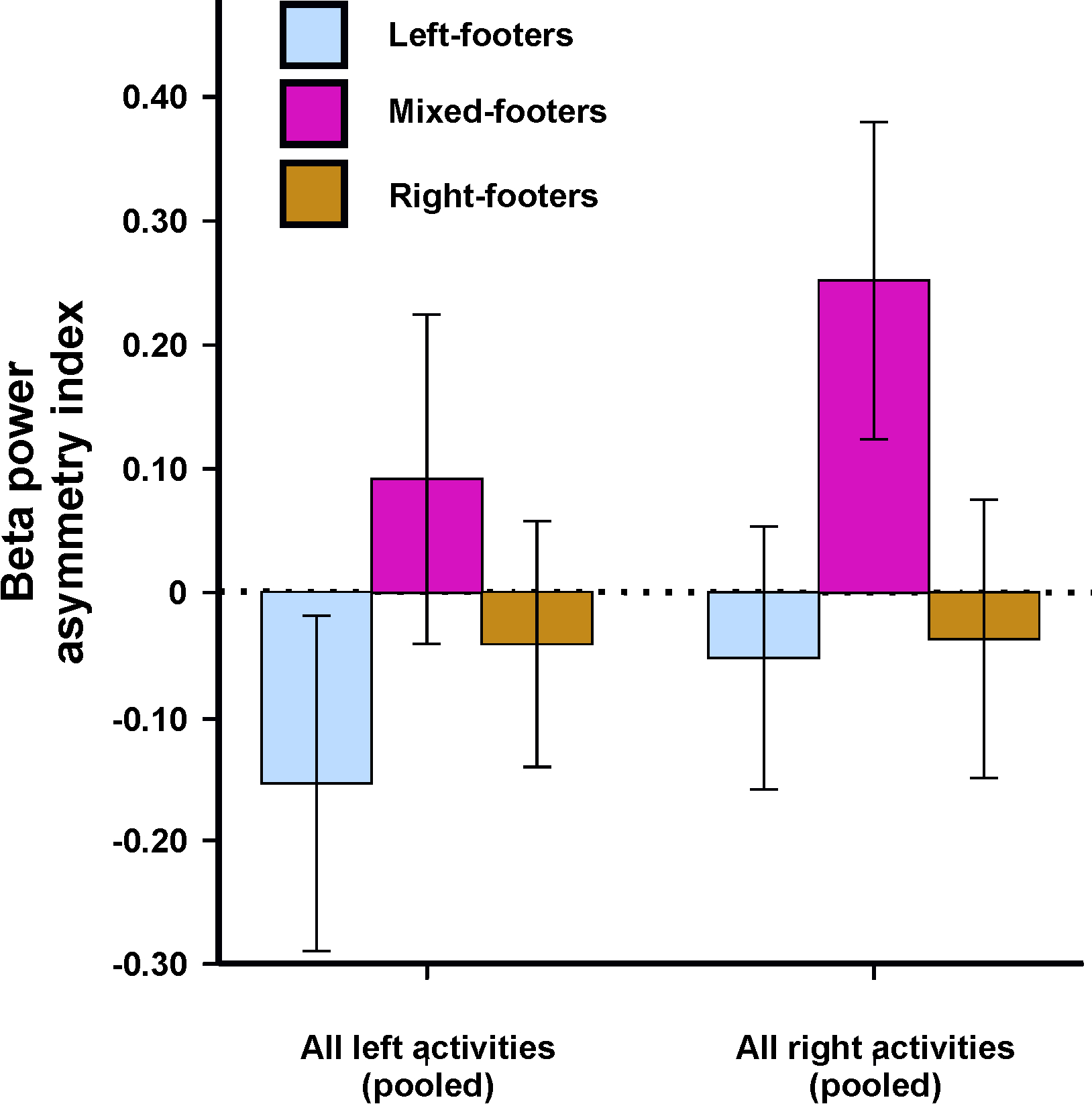

Supplement: FIGURE S5 — Beta power asymmetries for all individual tasks of the WFQ for left-, mixed-, and right-footers during left and right task performance. Error bars represent ± 1 SEM. [file Image_5.jpeg]

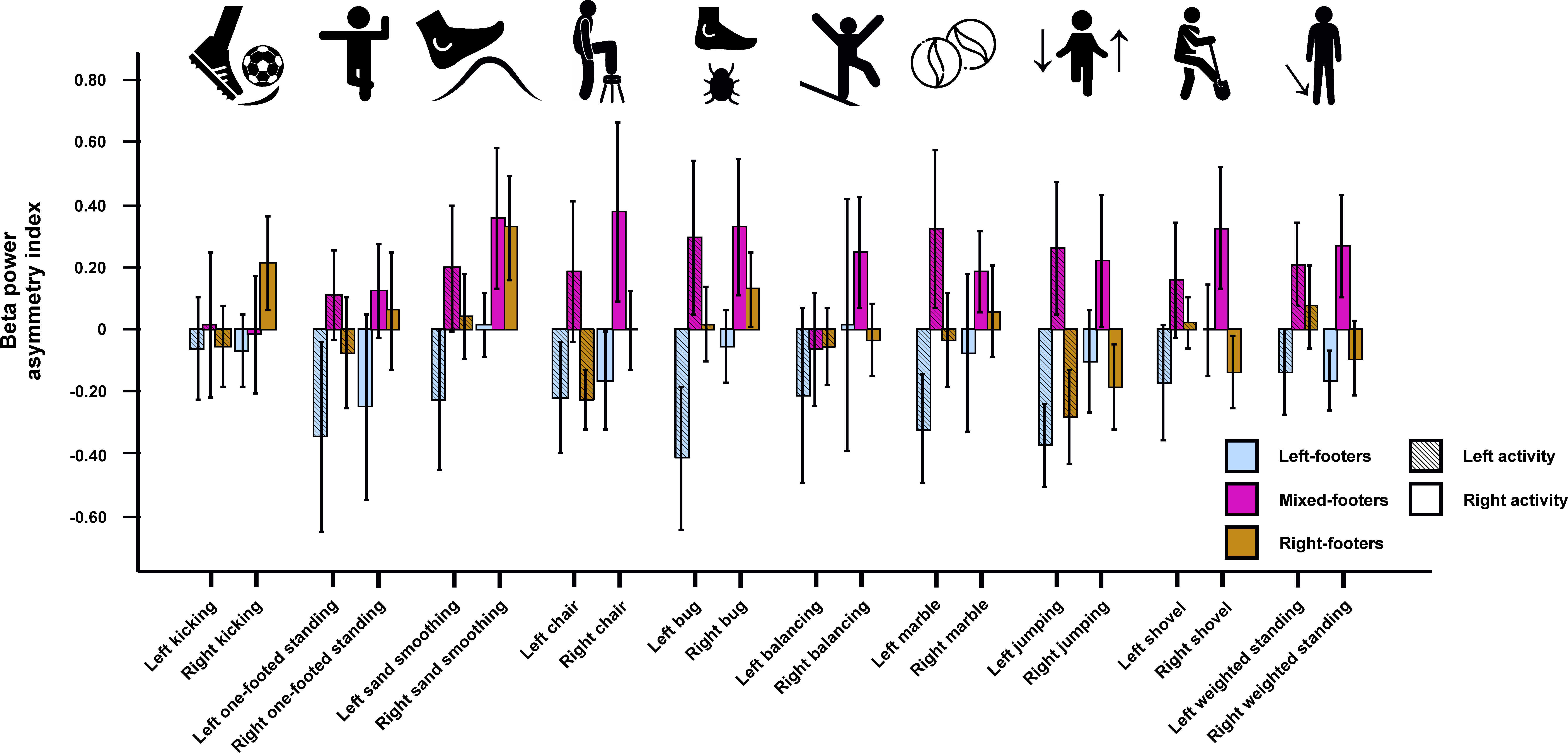

Supplement: FIGURE S6 — Beta power asymmetries for all individual tasks of the WFQ for left-, mixed-, and right-footers during left and right task performance. Error bars represent ± 1 SEM. [file Image_6.jpeg]
